# Multiple-Monitor HPLC Assays for Rapid Process Development, In-Process Monitoring, and Validation of AAV Production and Purification

**DOI:** 10.3390/pharmaceutics13010113

**Published:** 2021-01-17

**Authors:** Pete Gagnon, Blaz Goricar, Nina Mencin, Timotej Zvanut, Sebastijan Peljhan, Maja Leskovec, Ales Strancar

**Affiliations:** BIA Separations, Sartorius Company, Mirce 21, 5270 Ajdovscina, Slovenia; Blaz.Goricar@biaseparations.com (B.G.); Nina.Mencin@biaseparations.com (N.M.); timotej.zvanut@biaseparations.com (T.Z.); Sebastijan.Peljhan@biaseparations.com (S.P.); Maja.Leskovec@biaseparations.com (M.L.); ales.strancar@biaseparations.com (A.S.)

**Keywords:** adeno-associated virus, AAV, HPLC, intrinsic fluorescence, extrinsic fluorescence, light scattering, empty capsids, full capsids, process development, in-process analysis, validation

## Abstract

HPLC is established as a fast convenient analytical technology for characterizing the content of empty and full capsids in purified samples containing adeno-associated virus (AAV). UV-based monitoring unfortunately over-estimates the proportion of full capsids and offers little value for characterizing unpurified samples. The present study combines dual-wavelength UV monitoring with intrinsic fluorescence, extrinsic fluorescence, and light-scattering to extend the utility of HPLC for supporting development of therapeutic AAV-based drugs. Applications with anion exchange (AEC), cation exchange (CEC), and size exclusion chromatography (SEC) are presented. Intrinsic fluorescence increases sensitivity of AAV detection over UV and enables more objective estimation of empty and full capsid ratios by comparison of their respective peak areas. Light scattering enables identification of AAV capsids in complex samples, plus semiquantitative estimation of empty and full capsid ratios from relative peak areas of empty and full capsids. Extrinsic Picogreen fluorescence enables semiquantitative tracking of DNA with all HPLC methods at all stages of purification. It does not detect encapsidated DNA but reveals DNA associated principally with the exteriors of empty capsids. It also enables monitoring of host DNA contamination across chromatograms. These enhancements support many opportunities to improve characterization of raw materials and process intermediates, to accelerate process development, provide rapid in-process monitoring, and support process validation.

## 1. Introduction

Rapid analytical feedback is a critical element for timely development of processes that yield clinical quality biological drugs. It plays an equally important role in documenting that established processes remain within specified control limits during validation and manufacturing. HPLC monitored by UV absorbance at 280 nm has contributed substantially to fulfillment of these roles with recombinant proteins like monoclonal antibodies. Relatively speaking, however, recombinant proteins are compositionally and structurally simple, typically present at high concentrations, and are the dominant components in cell culture harvests.

Development of HPLC assays for adeno-associated virus (AAV) must accommodate a more challenging reality. Individual AAV capsids comprise 60 capsid proteins of 3 types, ideally but often not in a ratio of 1:1:10; which may lack encapsidated DNA or contain DNA of varying lengths, with varying proportions of single-stranded and double-stranded sequences [1,2,3]. Post-translational modifications impose additional layers of heterogeneity [3,4,5]. AAV cell harvests and lysates bear contaminant species and levels similar to those encountered with recombinant protein cell culture harvests but AAV levels are orders of magnitude lower, leaving AAV virtually a trace component in an overwhelming excess of contaminants [6].

Monitoring HPLC assays simultaneously for multiple parameters provides more sensitive, more accurate, and more insightful characterization of sample composition without compromising the ability to rapidly process large numbers of samples. The simplest case exploits dual wavelength monitoring. Nucleic acids absorb UV maximally at 260 nm and proteins at 280 nm. The ratio of absorbance at 260 nm and 280 nm is generally accepted as being about 1.8 for double-stranded DNA and about 2.1 for single-stranded species. The 260/280 ratios for empty and full AAV capsids in a non-chromatographic study were reported to be 0.59 and 1.44, respectively [7]. AEC has been shown to separate empty and full capsids into separate peaks for serotypes 1, 2, 4, 5, 6, 8, and 9 [6,8,9,10,11,12,13,14,15,16,17]. Empty capsid peaks separated from full capsid peaks by anion exchange (AEC) commonly exhibit ratios in the range of 0.6 to 0.7, while full capsids peaks commonly exhibit ratios in the range of 1.3–1.4. Intermediate ratios are often interpreted to indicate co-elution of empty and full capsids but they can also result from variation in the amount of encapsidated DNA among different capsid subpopulations, and/or from coelution of contaminating host cell proteins and/or nucleic acids.

UV profiles are unsuitable for judging the relative areas of dominantly-empty capsid peaks and dominantly-full capsid peaks directly from chromatograms. DNA absorbs about three times more UV per unit mass than capsid proteins and causes the proportion of full capsids to be overestimated [7]. Calculations incorporating extinction coefficients can be used to derive the amount of protein in each peak and then compare them [7,18], but this suspends the simplicity of estimating empty and full capsid proportions directly from relative peak areas. Even after adjustment for extinction coefficients, the dominance of UV absorbance by DNA reduces relative sensitivity for detection of empty capsids, especially if they are present at low levels [7]. Wavelength ratios are also used to estimate the distribution of host cell DNA contamination across chromatograms [6] but they tend to overestimate it. RNA also persists in cell harvests and has nearly identical spectral properties [19].

In-line monitoring of intrinsic fluorescence increases sensitivity over UV detection of proteins and minimizes the error factor imposed by nucleic acids. Intrinsic protein fluorescence derives mostly from tryptophan residues, with a much lesser contribution by tyrosine and still less by phenylalanine [20,21,22]. Tryptophan is remarkably abundant in AAV capsids proteins, constituting 2.2% of complete capsids [23]. VP1 from AAV2 contains 15 tryptophan residues. VP2 and VP3 each contain 12. The excitation and emission wavelengths of DNA overlap with tryptophan but intrinsic fluorescence emission of DNA is comparatively negligible [24,25,26]. This enables protein-based estimation of relative empty and full capsid peak areas directly from AEC chromatograms [6]. As with UV, intrinsic fluorescence-based estimates are potentially influenced by co-elution of empty and full capsids and/or by co-elution with contaminating proteins.

The high tryptophan content of AAV capsids also elevates their intrinsic fluorescence above the majority of cell culture and host cell proteins. Bovine serum albumin, which is only 7% larger than VP3, contains only two tryptophan residues (0.3%) [27]. Histone proteins have none [28,29,30,31,32,33]. Low histone fluorescence aids detection of AAV against high backgrounds of chromatin contamination across chromatography profiles of AAV lysates [6]. Tryptophan fluorescence is quenched to a degree by interactions with DNA [34]. The magnitude of such effects with full AAV capsids remains to be quantified. Intrinsic fluorescence also varies as a result of protein conformational changes [35]. This could alter intrinsic fluorescence to mass ratios under the extreme conditions commonly employed by affinity and ion exchange chromatography of AAV.

Sensitivity of nucleic acid measurement can be amplified by monitoring extrinsic fluorescence conferred by intercalating dyes [36,37,38,39,40,41]. These dyes pass through lipid membranes and provide a tool to assess titer according to the amount of DNA or RNA fluorescence [42,43,44,45]. Data are divided on their ability to enter proteins capsids. They are reported to do so with adenovirus [46] but not with AAV [47]. Results can be obtained quickly, but co-stained host cell DNA in the form of degraded chromatin inflates the fluorescence signal [6,18,43,44,45,46,47]. Chromatin interference can be factored out by measuring dye fluorescence before and after capsid lysis but with a net result of low sensitivity [47]. HPLC reduces chromatin interference by separating the virus from most of the chromatin and increases sensitivity by concentrating the virus [6,44,45].

Accurate determination of DNA mass by extrinsic fluorescence requires experimental controls to accommodate many interfering influences [37,38,39,40]. Single-stranded DNA sequences bind intercalating dies differently than double-stranded sequences. Co-staining of RNA imposes potential error. Nucleic acid-binding proteins interfere with dye access, depress uptake kinetics, and reduce sensitivity. Slow dye uptake can be overcome with extended incubation, but differing salt and divalent metal cation concentrations may introduce further variation. Photobleaching of the dye during staining and analysis can reduce fluorescence yield.

Chromatographic monitoring by light scattering represents an emerging area of interest. Early studies with protein characterization have focused on SEC [48,49,50,51,52] for two reasons. The first is that the chemical environment is constant across the elution profile. That ensures a constant refractive index, which is important for precision measurements [53]. The second is that light scattering by itself cannot discriminate size diversity within mixed samples. SEC separates by size and makes it possible to compare the amount of light scattering among bodies of different sizes. Larger particles produce more scatter than an equal mass of smaller particles. More recent publications show that light scattering detection combined with ion exchange chromatography produces useful information beyond the ability of SEC, despite the chemical environment changing across gradient elution profiles [52,54,55].

In either case, light scattering enables selective detection of AAV capsids from complex chromatograms [6]. Their 20–25 nm diameter provides good sensitivity. The size similarity of empty and full capsids makes it possible to estimate the relative proportions of dominantly-empty peaks and dominantly-full capsid peaks. However, full capsid peak area is inflated by the greater mass per full capsid [1,54,55]. Interference by proteins and other small host contaminants is negligible but chromatin contamination interferes with quantitative estimation in unpurified samples. Unfiltered cell cultures contain large amounts of chromatin remnants ranging in size from a few nm up to several µm [56]. Filtration to 0.45 µm truncates the top end of the range to about 400 nm but the remainder still overlaps with AAV [6,57,58,59,60]. Beyond simple monitoring of light scattering intensity to identify AAV peaks, light scattering offers potential to accurately determine AAV capsid size and mass in purified preparations.

This report documents the practical value of HPLC-based AAV analysis with multiple monitors. Examples include AEC, CEC, and SEC with samples at different process stages monitored by UV absorption at 260 nm and 280 nm by intrinsic fluorescence, extrinsic Picogreen fluorescence, and light scattering. Discussion follows, addressing interpretation and utility of these assays for qualification of raw materials, process development, process validation, and in-process monitoring of AAV manufacturing operations.

## 2. Materials and Methods

### 2.1. Samples and Sample Preparation

Triton X-100 lysates of sf9/BEV and HEK293 cells producing AAV2/8 were obtained from The University of Nantes, Center for Translational Therapy for Genetic Diseases, INSERM UMR 1089, Nantes, France. AAV8 was chosen particularly because AEC is known to separate empty and full capsids into separate peaks [6,9,10,16]. All were membrane filtered to 0.45 µm. Host cell DNA (chromatin) contamination was reduced in some by tangential flow filtration (TFF, 300 kDa membrane) into 20 mM Tris, 500 mM sodium chloride, 5 mM magnesium chloride, 1% sucrose, 0.1% Poloxamer 188, pH 8.0; they were then digested with a salt-tolerant nuclease enzyme (Kryptonase, 50 unit/mL, BIA Separations, Ajdovscina, Slovenia) at ambient temperature for 16 h in the TFF unit, and then processed further by TFF to remove nucleotides and nucleoproteins liberated by DNA lysis. Chromatin contamination was alternatively reduced by addition of prototype positively charged particles (CIMasphere H-Bond, BIA Separations) at a proportion of 2% (*v:v*), incubated mixing for 60 min, then filtered through a 0.45 µm membrane to remove solids.

Initial AAV purification was performed by CEC on 1 mL or 8 mL CIMmultus SO3 monoliths (BIA Separations). CEC columns were equilibrated to 50 mM formic acid, 200 mM sodium chloride, 1% sucrose, 0.1% Poloxamer 188, pH 3.5, eluted with a linear gradient to 50 mM formic acid, 2 M sodium chloride, pH 3.5, then cleaned with 2 M sodium chloride plus 1 M sodium hydroxide. Volumetric flow rate on 1 mL monoliths was 5 mL/min (5 column volumes (CV)/min). Volumetric flow rate for 8 mL monoliths was 40 mL/min.

Picogreen incubation time was evaluated on CEC-purified AAV with 2 µL of dye per 100 µL of sample at time intervals up to 24 h. In a separate series of experiments, the effect of Picogreen concentration was evaluated with 10 min staining at dye dilutions up to 500-fold. Samples were analyzed by SEC in 50 mM MES, 150 mM sodium chloride, 0.05% Poloxamer 188, pH 6.5, or with a variant buffer substituting 1 M sodium chloride in place of 150 mM. Total Picogreen signal was expressed as fluorescence peak area in mV*sec. Based on the results from these experiments, Picogreen staining of experimental samples was subsequently performed by adding 2 µL of Picogreen per 100 uL of sample, then incubating for 24 h at 4 °C in the dark (foil-wrapped) to minimize photobleaching of the dye. Dark autosampler vials were used for the same purpose.

Capsids were lysed in some experiments to release encapsidated DNA. CEC-purified AAV was diluted 5-fold in 50 mM MES, 150 mM NaCl, 0.05 M Poloxamer 188, pH 6.5. Proteinase K (Thermo Fisher Scientific, Waltham, MA USA) was added to a final concentration of 200 µg/mL and incubated at ambient temperature. Samples were analyzed at time intervals ranging from 1 h to 24 h.

Relative intrinsic fluorescence of tryptophan and calf thymus DNA (Sigma-Aldrich, Saint Louis, Mo USA) were compared by injecting 100 µL of tryptophan at a concentration of 10 µg/mL (total 1 µg) onto an analytical SEC column and determining the peak area in mV*sec. That value was compared to the peak area from a 100 µL injection of calf thymus DNA (Sigma-Aldrich) at 100 µg/mL (total 10 µg). The different sample concentrations were required to keep the peaks on scale during SEC.

Beyond the use of UV wavelength ratios, the respective identities of dominantly-full capsid peaks and dominantly-empty capsids peak separated by AEC were confirmed by PCR [61].

### 2.2. HPLC Analysis

Chromatographic analyses were performed on a PATfix LPG HPLC system with integrated MWD UV–Vis detector (190–700 nm, 8 channel deuterium lamp, 50 mm flow cell path length), conductivity, and pH monitor (BIA Separations); further equipped with a Prominence RF-20A (200–650 nm, dual wavelength fluorescence detector (Shimadzu, Kyoto, Japan), and a DAWN HELEOS II multi-angle light scattering detector (Wyatt Technology, Dembach, Germany).

UV absorbance was monitored at 260 nm and 280 nm. Light scattering was measured at an angle of 90°. Intrinsic fluorescence was monitored at an excitation wavelength of 280 nm and an emission wavelength of 348 nm. Extrinsic (Picogreen) fluorescence was monitored at an excitation wavelength of 485 nm and an emission wavelength of 520 nm.

Analytical SEC was conducted using a TSKgel GSW4000swxl column (7.8 mm × 300 mm, Tosoh Bioscience, Greisheim, Germany) with 50 mM MES, 150 mM sodium chloride, 0.05% Poloxamer 188, pH 6.5 at a volumetric flow rate of 0.5 mL/min.

Analytical CEC was conducted on a 100 µL CIMac SO3 monolith (BIA Separations). The column was equilibrated with 50 mM acetic acid, pH 4.0; eluted with a linear salt gradient to 50 mM acetic acid, 3.0 M sodium chloride, pH 4.0; then cleaned with 2 M sodium chloride plus 1 M sodium hydroxide. Volumetric flow rate was 1 mL/min.

Analytical AEC separations of empty and full capsid samples were performed on a 100 µL quaternary amine monolith (CIMac AAV, BIA Separations). The column was equilibrated with 50 mM bis-tris-propane, 2 mM magnesium chloride, pH 9.0; eluted with a linear salt gradient to 50 mM bis-tris-propane, 2 mM magnesium chloride, 200 mM sodium chloride, pH 9.0; then cleaned with 2 M sodium chloride plus 1 M sodium hydroxide. Volumetric flow rate was 1 mL/min. 

Analytical AEC analysis was alternatively performed using a 100 µL CIMac PrimaS monolith. The column was equilibrated with 10 mM bis-tris-propane, 10 mM Tris, 2 mM magnesium chloride, pH 8.0; eluted with a linear gradient to 10 mM bis-tris-propane, 10 mM Tris, 2 mM magnesium chloride, pH 10.0; then cleaned with 0.5 M phosphoric acid, 0.5 M acetic acid, and 0.05% benzyl alcohol.

## 3. Results

### 3.1. In-Line HPLC Monitoring of Empty and Full AAV Capsids

**Figure 1:**
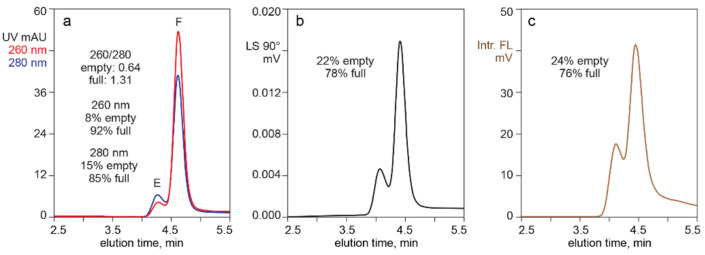
Separation of empty and full capsids by anion exchange (AEC) with different monitoring methods. (**a**) UV absorption at 260 nm and 280 nm. (**b**) Light scattering. (**c**) Intrinsic fluorescence.

Figure 1 illustrates separation of empty and full capsids by AEC with a salt gradient, monitored for UV absorbance, light scattering, and intrinsic fluorescence. The ratio of UV absorbances at 260 nm and 280 nm enabled identification of an early peak populated dominantly by empty capsids (260/280 = 0.64), and a later peak populated dominantly by full capsid (260/280 = 1.31). Integration of the UV elution profile at 260 nm estimated that the full capsid peak represented 92% of the total peak area. Absorbance at 280 nm estimated a full capsid peak area of 85%. Light scattering estimated that the full capsid peak represented 78%, but that should be understood to be an overestimate due to the influence of higher mass in full capsids [1,54,55]. Intrinsic fluorescence gave the lowest estimate of full capsid peak area at 76%. Even that should be understood to represent an overestimate, since the 260/280 ratio of the full capsid peak suggested that it contained a proportion of empty capsids. UV and light scattering-based estimates bear the same limitation.

In a separate fluorometric comparison of intrinsic fluorescence on SEC, DNA produced a peak area of 4.3 mV*sec per µg. Tryptophan gave an area of 38,943 mV*sec per µg, producing a differential of 9057 versus DNA. To provide a rough estimation of how much DNA contributes to the intrinsic fluorescence of full capsids, that differential was divided by 45.5 to account for AAV capsids containing 2.2% tryptophan. This produced a differential of 199 for equivalent masses of DNA and capsid proteins. Protein mass per capsid was calculated at 3.89 MDa (5 × 87 kDa (VP1) + 5 × 72 kDA (VP2) + 50 × 62 kDa (VP3)). DNA mass per capsid was calculated at about 1.5 MDa based on 5000 bases of single-stranded DNA with an average molecular weight of 303.7 Da per base. This yielded a full capsid protein to DNA mass ratio of 2.6 to 1. Multiplying the mass ratio times the intrinsic fluorescence differential per unit mass produced a per-capsid intrinsic fluorescence differential of 517. On this basis, capsid proteins would account for 99.8% of intrinsic full capsid fluorescence. Encapsidated DNA would contribute 0.2%.

### 3.2. In-Line HPLC Monitoring of Nucleic Acids

**Figure 2:**
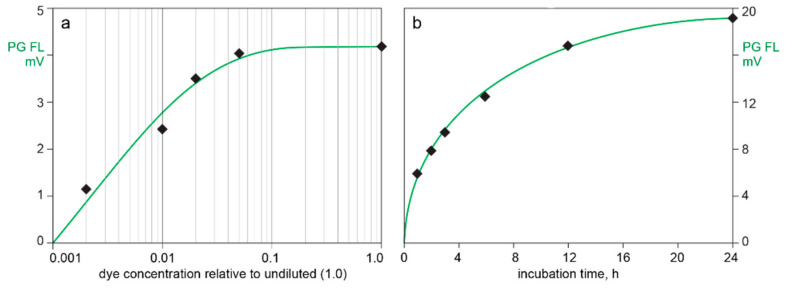
Picogreen staining requirements for adeno-associated virus (AAV) capsids. (**a**) Dye concentration. (**b**) Incubation time.

Figure 2 illustrates the effects of Picogreen dilution and incubation time on DNA staining of CEC-purified AAV in conjunction with SEC. Injection of Picogreen by itself produced no fluorescence peaks. A dye dilution of 20× gave a stained peak area of 96% compared to the undiluted dye. A dilution of 50× gave 75% and 100× gave 58%. Dye uptake at a dilution of 50× began to approach saturation after 24 h incubation. Picogreen fluorescence at 1 h was 31% of the value observed at 24 h. Formulating the SEC buffer with 1 M sodium chloride reduced the Picogreen fluorescence peak area by about 60% (not shown).

**Figure 3:**
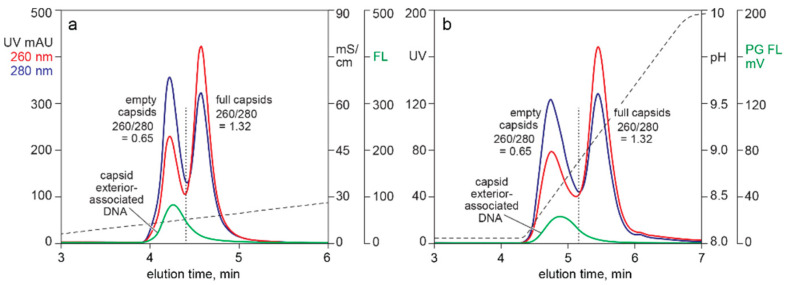
Picogreen detection of capsid exterior-associated DNA. (**a**) AEC eluted with a salt gradient. (**b**) AEC eluted with a pH gradient.

Figure 3 compares UV and Picogreen fluorescence for AAV capsids fractionated by two different AEC methods. The sample for both was CEC-purified AAV following nuclease-TFF treatment to minimize chromatin content. Both methods showed about 80% of the Picogreen signal associated with the empty capsid peak. The 260/280 ratio of the full capsid peak indicated the presence of empty capsids. This pointed to a likelihood that the Picogreen fluorescence in the full capsid peak was associated with co-eluting empty capsids, leading to a preliminary conclusion that Picogreen did not enter full AAV capsids.

**Figure 4:**
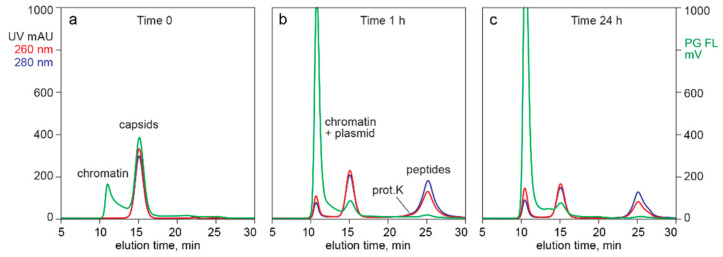
SEC of cation exchange (CEC)-purified AAV capsids stained with Picogreen. (**a**) Before treatment with proteinase K. (**b**) After 1 h incubation. (**c**) After 24 h.

Figure 4 provides additional evidence that capsid-associated Picogreen staining did not represent encapsidated DNA. Profile (a) illustrates the SEC Picogreen profile of CEC-purified AAV from filtered lysate. The exclusion limit of this column was at about 10 min. The Picogreen signal from 10.0 min to 13.5 min represented high molecular weight chromatin remaining after the CEC step. The peak centered at 15.5 min represented AAV capsids.

Digestion for 1 h with proteinase K (profile b) reduced the amount of capsids by about 32% but liberated a very large fraction of plasmid DNA. If the Picogreen fluorescence associated with the untreated capsids had been from encapsidated DNA, then the fluorescence area of the plasmid peak should have been 32% of the fluorescence peak area of the undigested capsids. Digestion also produced a late-eluting population of small peptides as expected, eluting immediately after a small proteinase K peak.

Digestion for 24 h liberated much more plasmid but surprisingly reduced the amount of capsids by only 55% compared with the undigested sample (profile (c)). The 260/280 ratio for capsids in the undigested control was 1.10. The ratio after 1 h was 1.10 and after 24 h was 1.12. These data indicate that empty and full capsids were digested at about the same rate. They also provided a third line of evidence that encapsidated DNA did not contribute to Picogreen fluorescence. If it had done so, then the 260/280 ratio of the undigested capsid peak should have been reduced in proportion to the increase in the size of the plasmid peak. Comparison of plasmid fluorescence with capsid fluorescence also highlighted that the amount of DNA associated with capsid exteriors represents a small fraction of the amount of DNA inside full capsids.

### 3.3. In-Line HPLC Characterization of Raw and In-Process Materials

**Figure 5:**
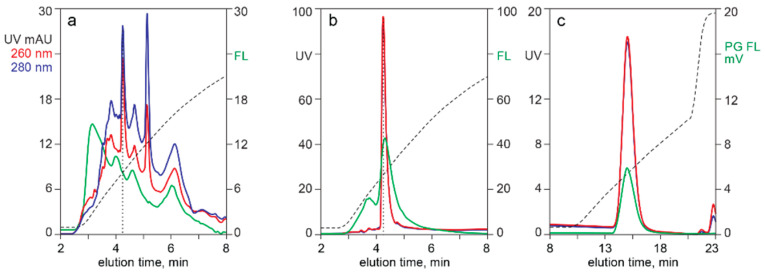
CEC with Picogreen staining for characterization of DNA contamination. (**a**) Clarified lysate. (**b**) After CEC capture from clarified lysate. (**c**) After CEC capture from lysate treated with nuclease and TFF.

Figure 5 illustrates the utility of analytical CEC for evaluation of raw materials and as a process development guide. Profile (a) shows the elution profile of filtered lysate pre-stained with Picogreen. The vertical dotted line on profile (a) indicates where AAV eluted, based on profile (b), which was run on the same column under identical conditions. The general parallel of 260 nm, 280 nm, and Picogreen fluorescence, all rising steeply at the beginning of the gradient then gradually sloping downwards, showed that chromatin dominated the contaminant population.

Chromatogram (b) shows CEC analysis after CEC capture from filtered lysate. Chromatogram (c) illustrates the CEC elution profile of AAV purified by CEC after the lysate was treated with nuclease and TFF to preemptively reduce chromatin contamination. Profiles (b) and (c) both show the 260 and 280 profiles virtually on top of each other. This is consistent with CEC eluting empty and full capsids in the same peak. The broader capsid peak on profile (c) is the result of a different gradient configuration on a 1 mL column. Profiles (a) and (b) were run on a 100 µL column.

The ratio of Picogreen fluorescence to UV absorbance at 260 nm in profiles (b) and (c) showed a clear benefit of preemptive chromatin reduction. The ratio of fluorescence to absorbance, measured as percent of full scale, provided an indication of relative DNA contamination. The ratio for the capsid peak in profile (b) was 0.47. The ratio for the capsid peak in profile (c) was 28% lower at 0.34. This could indicate that advance TFF-nuclease treatment reduced the amount of DNA bound to capsid exteriors but it could also indicate that the capsids in profile (b) co-eluted with chromatin. The latter hypothesis is favored by the additional observation that advance chromatin extraction (c) eliminated the DNA populations flanking the AAV peak (b).

**Figure 6:**
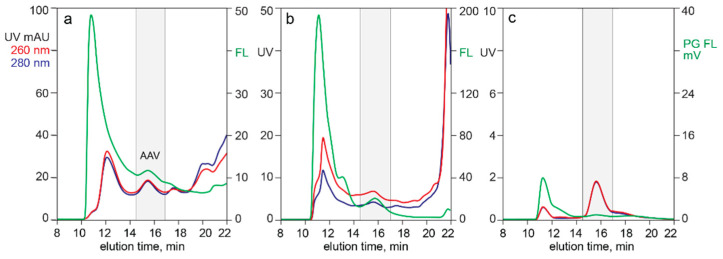
SEC with Picogreen staining. (**a**) Filtered sf9/BEV lysate. (**b**) Filtered HEK293 lysate. (**c**) Filtered HEK293 lysate after treatment with CIMasphere H-Bond particles.

Figure 6 illustrates the utility of monitoring analytical SEC with Picogreen fluorescence for evaluation of raw materials and as a process development guide. Profile (a) was developed from an AAV8 lysate produced in sf9/BEV cells. Profile (b) was developed from an AAV8 lysate produced in HEK293 cells transfected with the same plasmids. Heavy chromatin contamination is implied in both by the dominance of the 260 nm profile and confirmed by the Picogreen response. Panel (c) illustrates chromatin reduction by sample treatment with positively charged particles. A well-defined AAV peak was apparent from the UV trace, largely devoid of host cell contaminants except for a persistent fraction of high molecular weight chromatin.

**Figure 7:**
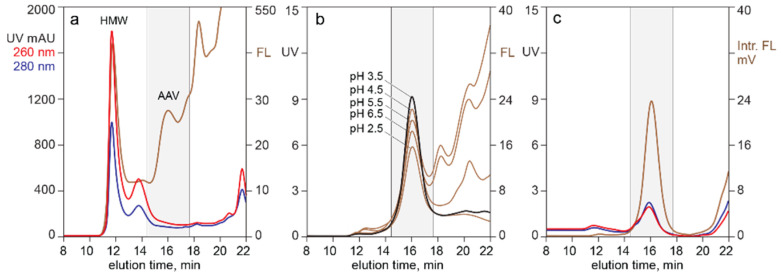
SEC with intrinsic fluorescence. (**a**) Filtered lysate. (**b**) After treatment with 4 M NaCl at indicated pH values. (**c**) After treatment with CIMasphere H-Bond particles at 4 M NaCl, pH 3.5.

Figure 7 highlights the value of monitoring analytical SEC with intrinsic fluorescence. The UV profile of the filtered harvest in profile (a) was so heavily dominated by chromatin that no AAV peak was apparent. Intrinsic fluorescence preferentially amplified the AAV signal because of its high tryptophan content and made it possible to visualize the capsids against a high background of contaminating protein fluorescence. Profile (b) illustrates overlaid intrinsic fluorescence profiles from a series of experiments performed on the filtered lysate shown in profile (a). Capsids were treated with 4 M NaCl at the pH values shown, then filtered to remove solids before SEC. Panel (c) illustrates results when the filtered lysate was treated with 4 M NaCl at pH 3.5 in the presence of positively charged particles.

**Figure 8:**
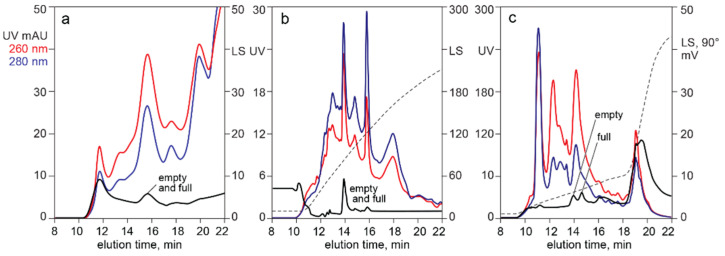
Light scattering identification of AAV in chromatograms of filtered lysate. (**a**) SEC. (**b**) CEC with salt gradient elution. (**c**) AEC with salt gradient elution.

Figure 8 shows the ability of light scattering to aid detection of AAV capsids in filtered lysates applied to analytical HPLC columns. Empty and full capsids eluted in the same peak with SEC (a) and CEC at (b). AEC separated them (c). The high light scattering background in (a) and (c) was caused by high molecular weight chromatin, also indicated by the dominance of the 260 nm profile across the chromatograms. Background light scattering was reduced across the salt gradient for CEC (b) because most of the high molecular weight chromatin remained bound during the salt gradient and required removal by NaOH [6]. This was also reflected in the dominance of the 280 nm profile. However, DNA contamination remains substantial, as shown in panel (a) of Figure 5.

## 4. Discussion and Conclusions

### 4.1. In-Line HPLC Monitoring by UV Absorbance and Intrinsic Fluorescence

Monitoring capsid separation by intrinsic fluorescence enables more accurate determination of empty capsid peak size versus full capsid peak size than UV because DNA contributes only 0.2% of full capsid fluorescence (Figure 1) [6,24,25,26]. This enables determination of relative peak sizes based almost exclusively on intrinsic fluorescence of capsid proteins. As with UV, partial co-elution of empty and full capsids or their co-elution with contaminating host proteins could affect accuracy.

The contribution of DNA could be significantly less than 0.2%. With tryptophan producing at least 9000 times more fluorescence per unit mass than DNA, even trace levels of protein contamination in a purified DNA control sample would substantially inflate apparent DNA content.

Monitoring intrinsic fluorescence also enhances sensitivity of AAV detection over host cell proteins by virtue of capsid proteins containing an unusually high proportion (2.2%) of tryptophan residues [23]. This particularly helps one visualize AAV in SEC chromatograms of unpurified lysates (Figure 6, Figure 7 and Figure 8). The other side of this coin is that intrinsic fluorescence underestimates contamination by proteins with lesser tryptophan content. This extends to underestimating chromatin contamination since histone proteins lack tryptophan [28,29,30,31,32]. The high proportion of tryptophan in AAV capsid proteins also contributes to their unusually high UV extinction coefficient of 3.72 at 280 nm [7].

### 4.2. In-Line HPLC Monitoring by UV Absorbance and Light Scattering

Light scattering enables easy detection and confirmation of capsids for all chromatography methods. With AEC of purified samples, the relative area of the dominantly-full capsid peak areas is slightly higher than that judged by intrinsic fluorescence but much more reasonable than the substantial overestimates derived from UV absorption (Figure 1). Light scattering with AEC can also provide insight about the relative size of empty and full capsid peaks in harvests and lysates (Figure 8). Co-elution with high molecular weight chromatin interferes with quantitative estimation, but light scattering can still be useful to evaluate large differences of empty and full capsids among different cell lines and transfection methods. Empty and full capsids co-elute in a single peak from SEC and CEC columns (Figure 5, Figure 6, Figure 7 and Figure 8). Measurement of light scattering intensity at a single angle is sufficient for capsid detection in crude and purified samples. Measurement of light scatter at multiple angles is necessary for accurate determination of particle size and mass in highly purified samples [47,48,49,50,51,52,53,54,55].

### 4.3. In-Line HPLC Monitoring by UV Absorbance and Extrinsic Fluorescence

Monitoring extrinsic fluorescence from intercalating dyes opens a wide range of opportunities to characterize nucleic acid content in AAV samples, but many issues require careful attention. The first is the long incubation time required to stain unpurified samples (Figure 2). Complete staining of purified DNA under optimal conditions requires only a few minutes, but longer incubation is required to overcome steric exclusion of the dye by strongly bound proteins in unpurified samples [36,37,38,39,40,41]. Protocols for determination of virus titer typically recommend an incubation period of 1 h [40,41,42]. This acknowledges suppression of dye uptake in unpurified samples but underestimates its magnitude. Figure 2 shows that 1 h incubation produces only 32% of the signal produced at 24 h. It also warns that variations of a few minutes more or less than 1 h would produce major errors.

The simplest application of extrinsic fluorescence is visualizing the distribution of host cell DNA in the form of chromatin across chromatograms of candidate purification methods (Figure 5 and Figure 6) [6,19]. Chromatin is a greater burden for AAV purification than for recombinant proteins because low AAV titers make the chromatin-to-product ratio tens to hundreds of times higher [6,19,57,58,59,60].

Figure 3, Figure 4, Figure 5 and Figure 6 demonstrate that Picogreen is effective in conjunction with AEC, CEC, and SEC, but quantitative comparisons should be made with care. Even assuming uniform staining conditions, chromatography conditions can displace intercalated dye. This is demonstrated by SEC in the presence of 1 M sodium chloride reducing Picogreen fluorescence by 60% compared to peak area in 150 mM sodium chloride. CEC purification of AAV commonly employs pH 3.5 and sodium chloride elution gradients up to 2 M. AEC commonly employs pH 9.0 and sodium chloride gradients up to 200 mM. AEC often employs divalent metal cations that are known to enhance Picogreen fluorescence [41]. The same factors that affect Picogreen fluorescence also affect composition and conformation of chromatin [19,58,62,63,64,65], which can further affect comparability among chromatography methods.

The ability of intercalating dyes to pass through lipid membranes makes them natural candidates to evaluate titers of lipid enveloped viruses [42,43,44,45], but they are apparently not able to enter AAV capsids [47]. Figure 3 and Figure 4 confirm the inability of Picogreen to detect AAV-encapsidated DNA but reveal DNA associated with empty capsid exteriors. Given that the samples in Figure 3 were treated in advance to remove chromatin, then partially purified by CEC, and finally fractionated by AEC, the association of empty capsids and DNA must be understood to be highly stable. Interactions of nucleic acids with nucleic acid binding proteins are known to exhibit such stability [57,58,59,60], but the phenomenon has not been previously reported for AAV capsids.

The fact that empty and full capsids coelute in SEC (Figure 6, Figure 7 and Figure 8) suggests that exterior-associated DNA is likely in the form of small fragments tightly bonded to empty capsid surfaces. Otherwise, they would increase the hydrodynamic diameter of empty capsids and cause them to elute earlier than full capsids. It also seems apparent that DNA binding to capsid surfaces must alter their native charge characteristics by neutralizing capsid positive charges involved in DNA binding. Large amounts of DNA per capsid might be expected to cause empty capsids to elute earlier from CEC than full capsids. On the contrary, empty and full capsids generally coelute from CEC in a single peak (Figure 5 and Figure 8).

More investigation is warranted to fully characterize the phenomenon of DNA-binding to capsid exteriors, further including how broadly it occurs across serotypes. Evaluation of highly purified empty and full capsids prepared by ultracentrifugation would be desirable to prevent chromatin contamination from confusing interpretation. Proteolysis of empty capsids to release the external DNA for analysis by other means is an obvious goal, but it will first require development of protocols for their quantitative lysis.

### 4.4. Applications of Multi-Monitor HPLC Assays

HPLC assays with multiple monitors create many opportunities to improve the quality of information to guide development, validation, and monitoring of production processes for AAV. Beyond supporting purification process development [6], they offer potential to compare productivity of different host cells, compare and optimize cell culture formulations, optimize transfection protocols, determine when to harvest, and develop sample preparation methods. After basic processes have been established, these assays can be used to set specifications and document that all aspects of the production chain proceed in compliance with those specifications.

HPLC also offers potential to document accuracy of other analytical methods or guide development of needed improvements. PCR is a good example. Accurate results require effective sample preparation methods. Sample preparation often involves treatment with proteolytic enzymes to liberate plasmid DNA from AAV capsids. However, Figure 4 shows that barely 30% of the capsids are lysed after 1 h and barely 50% after 24 h. It stands to reason that deficient lysis must affect apparent titers. Nuclease enzymes are often used in PCR sample preparation with the intention to eliminate non-encapsidated plasmid, which is then followed by a step to remove or inactivate the enzyme to prevent digestion of the plasmids [61]. If that step is less than 100% effective, loss of plasmid DNA will depress the apparent titer. Multi-monitor HPLC can be applied either to obtain confidence that sample preparation steps perform as intended or provide a timely warning and the analytical tools to develop that confidence.

The same is also true in reverse: other analytical methods can be used to interpret and validate the findings of HPLC-based assays. Co-characterization of empty and full capsid content by HPLC and cryo-TEM is an obvious example. HPLC characterization of full, partially full, and empty capsids fractionated by density gradient centrifugation is another. Either way, and whatever the analytical goal, complementarity among different analytical approaches increases the depth of insight they can provide.

## Data Availability

Data are contained within the article.
